# Successful Treatment of an Opium Habit, Morphia, Hypodermically, of Nearly Seven Years Standing

**Published:** 1874-12

**Authors:** J. B. Mattison

**Affiliations:** Chester, N. J.


### SUCCESSFUL TREATMENT OF AN OPIUM HABIT, MORPHIA, HYPODERMICALLY, OF NEARLY SEVEN YEARS DURATION.

BY J. B. MATTISON, M. D., CHESTER, N. J.

*(Read before the Meigs and Mason Academy of Medicine, of Middlefort, Ohio, and ordered published.)*

We appear, once again, before the profession with the closing details of a case of more than ordinary interest, which, on two occasicns, has been presented to the fraternity; once* detailing the patient's initial history and proposed plan of therapeutical operations looking to his disenthrallment from a most debasing servitude of mind and body, and, subsequently,† citing the indifferent success attending our effort, owing, in much measure, to circumstances beyond control.

We have now the pleasure of placing on record the complete success crowning a second endeavor; and, as it contains several features of professional interest, and bearing somewhat on the mooted question of sudden or gradual withdrawal, we opine its presentation will not be unacceptable.

Charles Dimond, whose history as an excessive consumer of morphia, hypodermically, was narrated through the columns of the *Medical and Surgical Reporter* (vide No. 821), applied to us December 18, 1873, for relief from the most atrocious attack of tic-douloureux we ever witnessed, utterly defying every remedial measure brought to bear upon it during several days medication, and which gave way finally, and we hope forever, to a single deep injection of chloroform after the manner first suggested and practiced by Professor Bartholow, of Cincinnati, and details of which we reported in full in the *N. Y. Medical Record* for May 1.

When we first made an attempt at this man's reformation, his average consumption of morphia was five grains per diem. After its failure, he returned to the practice of his baneful habit more ardently than ever, soon doubling his former quantity; and when

* Medical and Surgical Record, No. 821.

† Ibid, No. 905.

he presented himself with his trifacial trouble, it was still larger, his weekly consumption being from one to two drachms, subcutaneously.

Notwithstanding the severity of his neurotic suffering, he expressed a desire to make, once more, an effort towards bursting the bonds that bound him, and, accordingly, passed into our possession his syringe; but the pain was so urgent such an attempt was not to be entertained for a moment.

For four days subsequently we essayed a variety of treatment looking to its relief, using the morphia in truly *heroic* doses, reaching as high as *five grains* at a *single* injection, with no apparent effect whatever; and only succeeded in putting a quietus upon it by the injection of chloroform, as before mentioned. This was followed in four minutes by a highly soporific condition, which continued for several hours, during which it occurred to us, could we prescribe anything which would have the effect of *prolonging* this state, we might make a break in upon his opium habit which would prove highly advantageous so far as an attempt at its withdrawal was concerned. Nothing suggested itself as more likely to accomplish this object than hydrate of chloral, for if it be true, as claimed, that the peculiar hypnotic virtues of this drug are due to the elimination of chloroform, certainly it would seem to be the most eligible agent, under the circumstances, inasmuch as it could but intensify and prolong the action of the chloroform subcutaneously administered. Accordingly, the patient was directed to bed, and chloral ordered, in scruple doses, every half hour, until its soporific properties were decided. Forty grains sufficed to procure a sleep of five hours duration. The same quantity—dose and interval as before—was again administered, when he fell into a profound slumber which continued for ten hours.

On awakening, he soon became clamorous for his morphine (an interval of nearly forty-eight hours having elapsed since an injection); but as we had determined to ascertain the degree of success attending a second attempt at its withdrawal, his demand was not acceeded to; but in lieu thereof, strych. sulph. gr. $\frac{1}{48}$ was injected hypodermically, ter die, with the impression on the part of the patient that the injection consisted of morphia in reduced amount, an idea of which, of course, we were exceedingly careful not to disabuse him. The dose of strychnia was given in *two* injections, for the quantity of liquid injected was an object of prime importance with him; and, as showing the effect of treatment purely psychical, it may be noted that, time and again, after giving him his usual allowance, and he was still unsatisfied. An injection of *aq. pur.*, clandestinely administered would exercise, seemingly, a wonderfully potent power for good, and quiet all his clamors for the time being at least.

Apart from the mental effect of the strychnia injections, the powerful tonic properties of this drug in toning up his nervous system after the prolonged opium debauch, and in eradicating a strongly marked neurotic taint were brought into play, and, we think, with signally good results.

In addition, we administered, subcutaneously, at bed-time, atrop. sulp. grains $\frac{1}{60}$ to $\frac{1}{30}$, according to the sciatic pain, restlessness and other varying circumstances of his condition, which yielded effects very satisfactory, and of which we shall speak more in detail hereafter.

Conjoined with this hypodermic medication, the patient was placed upon the use of the following prescription: ℞. Magnes. sulphas, ℥ss; mangan. sulph. ʒij; fer. sulph. ℈ij; quin. sulph. ℈j; acid sulph. dil. ℥ss; aq. ad. ℥viij. M. S. Tablespoonful in wineglass of water, *ter die,* and a nourishing diet—beef tea especially—ordered.

To procure sleep, chlor. hyd. was prescribed in a minimum dose of grs. xxx, and this quantity pushed grs. x at a dose as often as required during the night, until the object aimed at was accomplished.

The above tonic was soon set aside, proving ineligible on account of its bulk, and Wyeth's elix. fer. quin. et strychnia substituted in ʒij doses (strych. gr. $\frac{1}{30}$) thrice daily, continuing strychnia and atropia, hypodermically, as before.

After using this tonic one week he was ordered: ℞. Quin. sulphas, gs. ij; tinct. fer. chlor. ʒss; liq. potass. arsen. gtts. x. M. S. Take at a dose, diluted, after each meal—the other remedies to be continued.

Such was the general plan of treatment pursued—a full, nourishing diet, with the most efficient of nerve-tonics, hypodermically and per. via. nat., and it culminated, after a persistent prosecution of *four weeks* duration, in a consummation as complete and satisfactory as could have been wished for.

Under the above regimen our patient gained strength rapidly; his appetite became actually voracious, and he increased in weight markedly, averaging a fraction less than five pounds per week. No less gratifying was the improved change in his *moral* demeanor. In fact, he seemed to undergo a complete transformation, so that, at the expiration of the time mentioned, "old things had passed away" and he stood forth a *new* man thoroughly disenthralled from a most degrading bondage, and fully freed from a physical infirmity which for a three-quarter score of years it had been his painful lot to endure.

A few remarks somewhat *in extenso* on some points connected with this case may not be altogether devoid of interest.

A most decided obstacle to the withdrawal of the morphia was the severe sciatic pain to which the patient had for fifteen years been subject, and which broke out in full fury soon after the relief of his trigeminal disorder. For the removal of this we employed various remedies, all of which failed us until we resorted to vesication of the main nerve involved, applying, simultaneously, no less than *fourteen* blisters about three-fourths of an inch square over the track of the nerve affected from the point of its emergence to the popliteal space. These were allowed to remain sufficiently long to produce superficial vesication, dressed with cerat. simp., and from that date the sciatic suffering ceased, as by magic, and absolutely failed to put in its appearance thereafter.

The rapid and radical relief from the cantharidal application was so notable that it deserves more than a passing notice. It confirms fully the observations of Cotugno, Valleix, Anstie and Stille, more especially the latter, who declares: "Sciatica, more than other forms of neuralgia, is rebellious to this and to all forms of treatment, yet it is more amenable to methodical blistering than to any other exclusive method whatever."

Cotugno, first to use cantharides as a counter-irritant for the cure of sciatica, got to himself a wide-spread reputation for the remarkable success attending his treatment, notwithstanding his etiological idea of the disease would scarcely be considered, at this day, correct. Valleix asserts that blisters hold the highest rank in the treatment of neuralgia. And Anstie, in his classical work on this disease, while not admitting them *universally* applicable, insists that they are of the *greatest possible* service in a *large* number of instances, and observes that, in sciatica, we sometimes "obtain immediate success by two or three repetitions of the flying blister somewhere over the trunk of the nerve." It must not be overlooked, however, that in order to reap this striking benefit the blisters must be "flying." Any action which exercises a depletory effect under the mistaken idea that the materies morbi can thereby be removed, will fail of its object; in fact, we should confidently anticipate a distinct aggravation of the sciatic suffering. Nor must it be forgotten that in the case under consideration, the patient had the benefit of what we believe to be the remedy, *par excellence*, for *obstinate* cases of sciatica, and we are by no means sure that in the *milder* cases a similar treatment would not speedily suffice for a cure, viz.: strychnia, *hypodermically* administered. And we give it as our *confident* opinion that the number of cases *utterly* unyielding will be *very limited* indeed, where a thorough trial of this drug, in gradually increasing quantity until moderate evidences of its toxic properties become apparent, is resorted to, conjoined with that degree of counter-irritation so strongly endorsed by Valleix, Stille, Anstie and others. It may be noted that in this instance, the strychnia was continued for four weeks, during one week of which the tri-daily dose was equivalent to *one-tenth* of a grain, before symptoms of strychnism manifested themselves, and then only to a *limited* extent.

Our patient, ignorant as to the nature of the strychnine injections, was cognizant of the atropia, which was employed with a three-fold object—its anodyne property, which, inferior as a rule to morphine, has, nevertheless, a marked power for good, a quality that was appreciated and taken advantage of by our subject.

Secondly, we had previously demonstrated to our entire satisfaction—*Vide Reporter, No.* 905—the notable influence of this drug in restraining excessive ephidrosial action. In this last instance, its reputation in this direction was still further enhanced. The observations of Ringer, especially, corroborated recently by Tidd, of Ohio, and others, leave no doubt as to the potency of belladonna, and especially its alkaloid in hyper-cutaneous secretion.

Again, in a previous use of it in this case, we found our patient rapidly relieved from an obstinately torpid intestinal condition, which was to him a source of much discomfort. The same state of affairs a second time presenting itself, the atropia fulfilled our expectation, its administration being soon followed by a return to normal evacuations. Trousseau claimed for belladonna, the remedy *par excellence*, in habitual constipation. He says it does not purge or produce loose stools, but only renders defecation easier. Our experience with it, in the case under observation, was strongly substantiative.

These two valuable properties of this drug, if properly taken advantage of, must largely extend the range of its therapeutical operation. Many pathological conditions will present themselves, in which one or the other of them may prove exceedingly beneficial. As to other effects produced by the dose we employed—gr. one-sixtieth to one-thirtieth—nothing beyond marked faucial dryness and moderate pupillary dilatation was manifested.

No ill results followed the chloral, though it was given in consecutive doses of thirty, forty, fifty and sixty grains, nightly.

Markedly wide is the diversity of opinion among professional men as to the propriety of sudden or gradual withdrawal of the habitual intoxicant in these cases of opium inebriation. In this instance no less an authority than Hillard Parker, at whose clinic this man was presented, advised his incarceration and the morphia shut off at once and forever. Sooner than submit to that, the patient repeatedly declared to us, he would have committed self-destruction. An attempt of the kind was made by the surgeon of a New York hospital, but it "came to grief," for the man became so violent, smashing windows and the like, that the hospital authorities were glad enough to get rid of him. Corroborative of Prof. P.'s advice, Howe asserts that "tapering off" will not result in cure.

Opposed to this opinion, among others, is Dr. Parrish, who, as superintendent of an asylum for inebriates, has a fine field for observation, and who declares that he has "no hesitation in expressing an opinion which has settled into a fixed judgment with me, that for the class of cases with which I have had to do," and we presume they were by no means unique, "the system of gradual reduction has answered so well that I cannot bring myself to adopt the other plan," and who gives cogent reasons therefor, and fortifies his statements by citing several cases where this plan has been followed by conspicuously good results.

In an address delivered by him before the "American Association for the Cure of Inebriates," the subject of "Opium Intoxication" is dwelt upon at length, and we refer our readers to a report of the same in the *Medical and Surgical Reporter* for November 15 and 22, 1873.

Among other deductions from his admirable remarks, are the following:

"To relieve the symptoms it is desirable to avoid the shock, as it is desirable to avoid it in surgical operations.

"For this purpose, the practitioner should immediately reduce the accustomed supply to the minimum dose, which will meet this condition.

"When the minimum is reached, the suffering of the patient begins, and then the practice should be to give tone to the nervous system as the opium stimulus is withdrawn. The reduction should be in minute quantities and the tonic doses full and persistent.

"The moral sentiment, the confidence and courage of the patient should at all times be kept up to the attainable degree.

"Such a course will almost always secure the desired result."

The above we believe to be the plan of treatment best adapted to a large majority of cases. True, we cannot support it from personal experience, by reference to a single case so managed; but it appeals itself (in our judgment) most strongly to "common sense," which is better than any theory, and, besides, has the indorsement of those whose *successful* experience entitles them to the very highest respect and belief.

From a superficial view, a plan of treatment directly the reverse of what we advocate answered a perfect purpose in the case under consideration, and, seemingly, our practice was rather inconsistent with our precept. Granted; but there were some unusual circumstances in this instance, which, fully explained, will make it far from inexplicable why we succeeded so well notwithstanding a complete and immediate withdrawal of the accustomed stimulus.

In the first place, one leading indication under the gradual dim -

nution regime—avoidance of shock—was carried out in a manner similar to its fulfillment in surgical operations, viz: by the production of anæsthesia. The injection of the chloroform was followed in four minutes by a condition quite akin to that resulting from its moderate inhalation, which condition was prolonged, to a greater or less degree, for days, by the free administration of chloral. That by this, the man's perception of a desire for his opiate was blunted, we have not the slightest doubt. And this effect being produced in the *early* stages of its withdrawal, when the suffering attending the same was *keenest*, swayed the chances in his favor more heavily than they would have been at any subsequent period.

It must not be inferred, however, when this state of partial anæsthesia was no longer deemed advisable, that the inbreak upon his baneful habit was sufficiently great to enable him to wage the yet remaining battle free from pain. Far from it. On the contrary, he suffered, psychically, to an extent inconceivable save by those "in bonds, as bound with him!" But having gotten thus far on his journey towards recovery, and feeling fully assured that if, by any means, he could be enabled to hold out a little longer, the goal would be reached, his importunate pleadings were unheeded, and, slowly but surely, his "*devil*," as he styled the horrible craving, loosed its hold until, just *twenty-one days* from the date of his last morphine injection, he shook it off altogether, and stood forth *victor!*

From the foregoing history it may safely be deduced that the opium habit, while throwing about its victims an almost irresistibly seductive influence, is one which *can* be eradicated by a proper course of therapeutical management, and the unhappy victims of this increasing vice restored to their pristine physical and psychical condition.

But we reiterate that, in our opinion, this *cannot* best be done by a sudden and sweeping withdrawal of the habitual intoxicant. This course, especially if it be appreciated by the patient, will be very apt to give rise to a spirit of insubordination quite fatal to the success of the movement, standing, as it inevitably would, in the way of keeping up the courage and coöperation to the desired degree.

Conjointly, everything necessitating a corporeal demand for the anodyne properties of the drug must be thoroughly removed.

Added thereto must be a hygienic circumstancing about the unfortunate devotee, aided by medication, which will tend, most efficiently, in every possible manner, to tone up the shattered nervous system and make amends for the setting aside of the accustomed inebriant.

Such a plan of procedure will, we believe, rarely fail of eventuating in the "consummation devoutly to be wished for."

